# Recurrent Tumor-Induced Osteomalacia After Surgical Resection: A Case Report Demonstrating Successful Treatment With Burosumab

**DOI:** 10.7759/cureus.114961

**Published:** 2026-08-22

**Authors:** Carla Nicolau, Miguel Bigotte Vieira, Joana Dinis Melo, Caixia Zhu, Lino Fonseca, João Calado, Ana Carina Ferreira, Cristina Jorge, Anibal Ferreira

**Affiliations:** 1 Nephrology, Unidade Local de Saúde de São José, Lisbon, PRT; 2 Rheumatology, Unidade Local de Saúde de São José, Lisbon, PRT; 3 Radiology, Unidade Local de Saúde de São José, Lisbon, PRT; 4 Neurosurgery, Unidade Local de Saúde de São José, Lisbon, PRT; 5 Nephrology, Hospital Curry Cabral, Centro Hospitalar Universitário de Lisboa Central (EPE), Lisbon, PRT

**Keywords:** burosumab, fibroblast growth factor-23, hypophosphatemia, osteomalacia, tumor-induced osteomalacia

## Abstract

Tumor-induced osteomalacia (TIO) is a rare paraneoplastic syndrome in which a mesenchymal tumor secretes fibroblast growth factor 23 (FGF-23), causing renal phosphate wasting and osteomalacia. A 62-year-old woman was referred to our hospital with a two-year history of progressive muscle weakness and generalized bone pain. The patient was nonambulatory and required a wheelchair for mobility. Laboratory evaluation revealed hypophosphatemia and low serum 1,25-dihydroxyvitamin D levels. Further investigation of the hypophosphatemia demonstrated an elevated fractional excretion of phosphate (FeP) and increased serum FGF-23 levels. Genetic testing was negative. A ^68^Ga-DOTANOC PET/CT scan identified an infiltrative cervical lesion with increased somatostatin receptor expression. The tumor was surgically resected, after which the patient recovered functional ambulation, and serum phosphate normalized. One year later, the patient reported recurrence of musculoskeletal symptoms. Laboratory findings showed hypophosphatemia with an elevated FeP, raising suspicion of tumor recurrence. A repeat ^68^Ga-DOTANOC PET/CT scan demonstrated increased somatostatin receptor expression in a right parietal calvarial lesion that was not amenable to surgical resection or radiotherapy. Initiation of burosumab therapy led to the normalization of serum phosphate levels and complete resolution of symptoms. TIO is a rare disorder that is frequently misdiagnosed as osteoporosis, resulting in substantial diagnostic delay. A systematic evaluation of hypophosphatemia is essential for timely diagnosis and appropriate management. This case supports burosumab as an effective therapeutic option for achieving biochemical and clinical improvement in patients with unresectable TIO.

## Introduction

Tumor-induced osteomalacia (TIO) is a rare acquired paraneoplastic syndrome caused by excessive secretion of fibroblast growth factor 23 (FGF-23) by phosphaturic mesenchymal tumors (PMTs). Excess FGF-23 reduces renal phosphate reabsorption and suppresses 1,25-dihydroxyvitamin D synthesis, leading to renal phosphate wasting, hypophosphatemia, and impaired bone mineralization. Patients typically present with progressive bone pain, proximal muscle weakness, fatigue, reduced mobility, and recurrent insufficiency or fragility fractures [1].

Advances in functional imaging, particularly somatostatin receptor-based positron emission tomography/computed tomography (PET/CT), have substantially improved tumor localization. Nevertheless, a proportion of tumors remain occult or arise in anatomically challenging locations that preclude complete surgical resection [2]. Complete surgical excision is the treatment of choice and is curative in most cases; however, recurrence occurs in approximately 7-16% of patients, usually within the first three years after surgery [3,4]. When the tumor is unresectable or cannot be localized, conventional treatment with oral phosphate and active vitamin D analogues is often limited by poor tolerability and long-term complications, including secondary hyperparathyroidism, hypercalciuria, and nephrocalcinosis [5,6].

Burosumab, a fully human monoclonal antibody targeting FGF-23, directly addresses the underlying pathophysiology of TIO by restoring renal phosphate reabsorption and increasing circulating 1,25-dihydroxyvitamin D concentrations [7]. Clinical studies have demonstrated sustained improvements in serum phosphate levels, fracture healing, physical function, pain, and health-related quality of life in adults with unresectable or nonlocalizable TIO, supporting its approval for this indication [8]. However, because TIO is exceptionally rare, evidence regarding the long-term effectiveness of burosumab remains limited, particularly in patients with recurrent unresectable disease following previous surgical resection. We report a case of recurrent unresectable TIO successfully treated with burosumab, resulting in sustained biochemical correction and marked clinical improvement. This case adds to the limited evidence supporting its long-term use in this challenging clinical setting.

## Case presentation

A 62-year-old woman was referred to our hospital in January 2022, with a two-year history of progressive generalized bone pain and proximal muscle weakness. Her symptoms began in 2020 with pain involving the lower limbs and pelvis. In 2021, she sustained an incomplete pelvic fracture and a left femoral metaphyseal fracture after a low-energy fall, both managed conservatively. She developed worsening pain, fatigue, and progressive muscle weakness, leading to severe functional impairment and loss of independent ambulation. During the course of her illness, the patient was initially misdiagnosed with osteoarthritis and received ineffective treatment with glucosamine and bilateral knee viscosupplementation. A subsequent diagnosis of osteoporosis prompted referral to the Rheumatology Department in February 2022. Physical examination revealed diffuse tenderness over the pelvis and lower limbs and symmetric pelvic girdle weakness. No palpable masses were identified. Dual-energy X-ray absorptiometry showed osteoporosis (total femur T-score, -3.2), and laboratory evaluation demonstrated severe hypophosphatemia, prompting referral to the Nephrology Department. Biochemical evaluation demonstrated renal phosphate wasting, evidenced by an inappropriately elevated fractional excretion of phosphate (FeP), together with hypophosphatemia, reduced 1,25-dihydroxyvitamin D levels, elevated parathyroid hormone and alkaline phosphatase concentrations, and increased serum FGF-23 levels (Table 1). 

**Table 1:** Longitudinal laboratory data before surgery and after burosumab initiation ALP: alkaline phosphatase; FGF-23: fibroblast growth factor 23; FeP: fractional excretion of phosphate; PTH: parathyroid hormone; SC: subcutaneous

	Initial result (before surgery)	Six weeks post burosumab initiation (20mg SC)	Reference range
Phosphorus (mg/dL)	1.5	3.8	2.5-4.9
FeP (%)	18.99	11	<5-20%
Calcium (mg/dL)	8.7	8.9	8.5-10.2
1,25 dihydroxyvitamin D (pg/mL)	<2.6	26	19.6-54.3
PTH (pg/mL)	101	43	15-65
ALP (U/L)	459	95	35-105
Creatinine (mg/dL)	1.1	0.9	0.6-1.3
FGF-23 (UA/mL)	348	85	<180

Genetic testing targeting the ADCY10, AGXT, AP2S1, CLCN5, CLDN16, CLDN19, CYP24A1, DMP1, ENPP1, FAM20A, FAM20C, FGF23, GNA11, GHRPR, HNF4A, HOGA1, HPRT1, KCNJ1, OCRL, PHEX, SGK3, SLC12A1, SLC22A12, SLC26A1, SLC2A9, SLC34A1, SLC34A3, SLC3A1, SLC4A1, SLC7A9, and SLC9A3RA genes identified no pathogenic variants, making a hereditary hypophosphatemic disorder less likely. TIO was therefore suspected, and oral phosphate and calcitriol were initiated while tumor localization was pursued.

**Figure 1:**
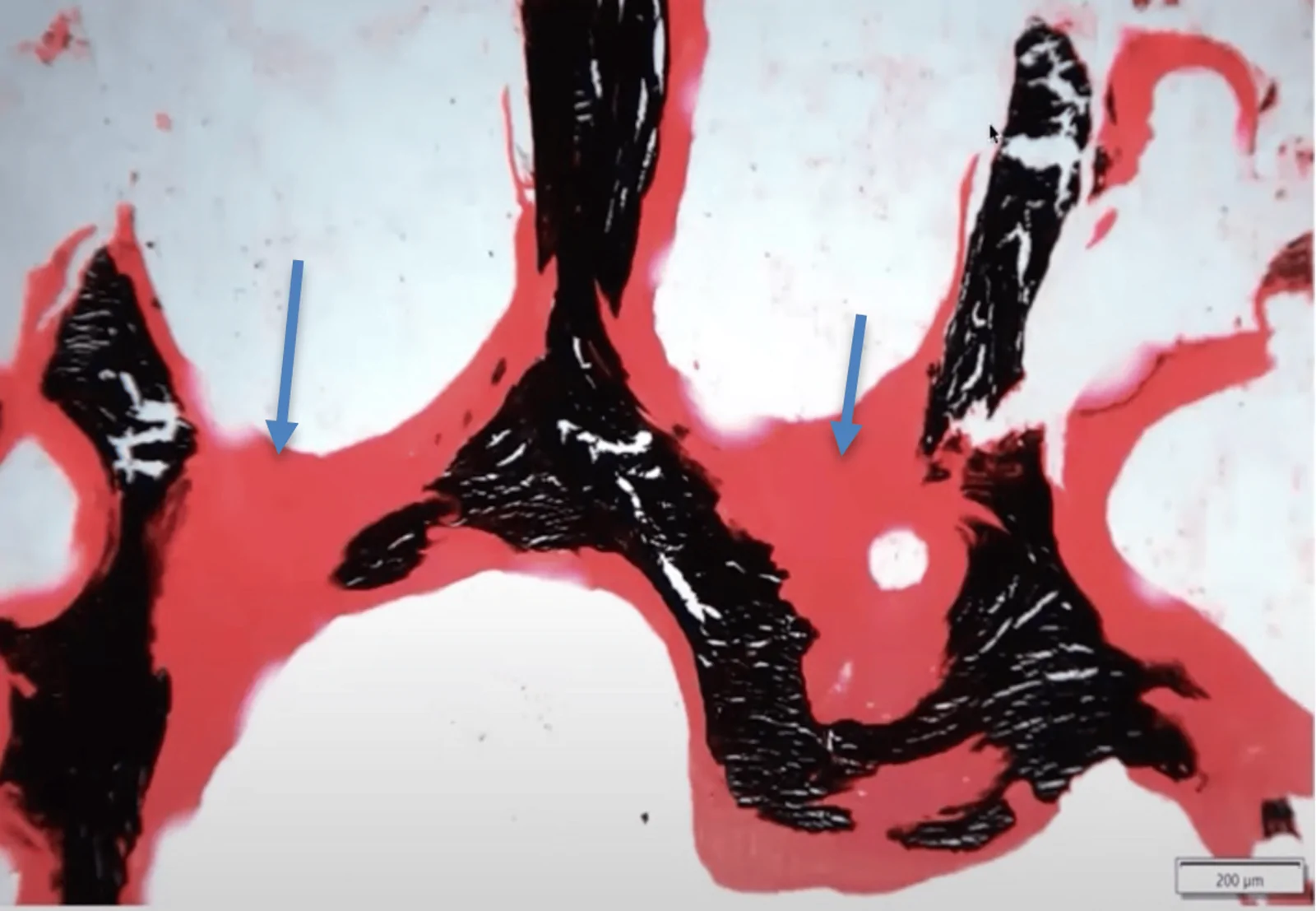
Bone biopsy revealing osteomalacia Von kossa staining, demonstrates increased osteoid (arrows). Original magnification x100

**Figure 2:**
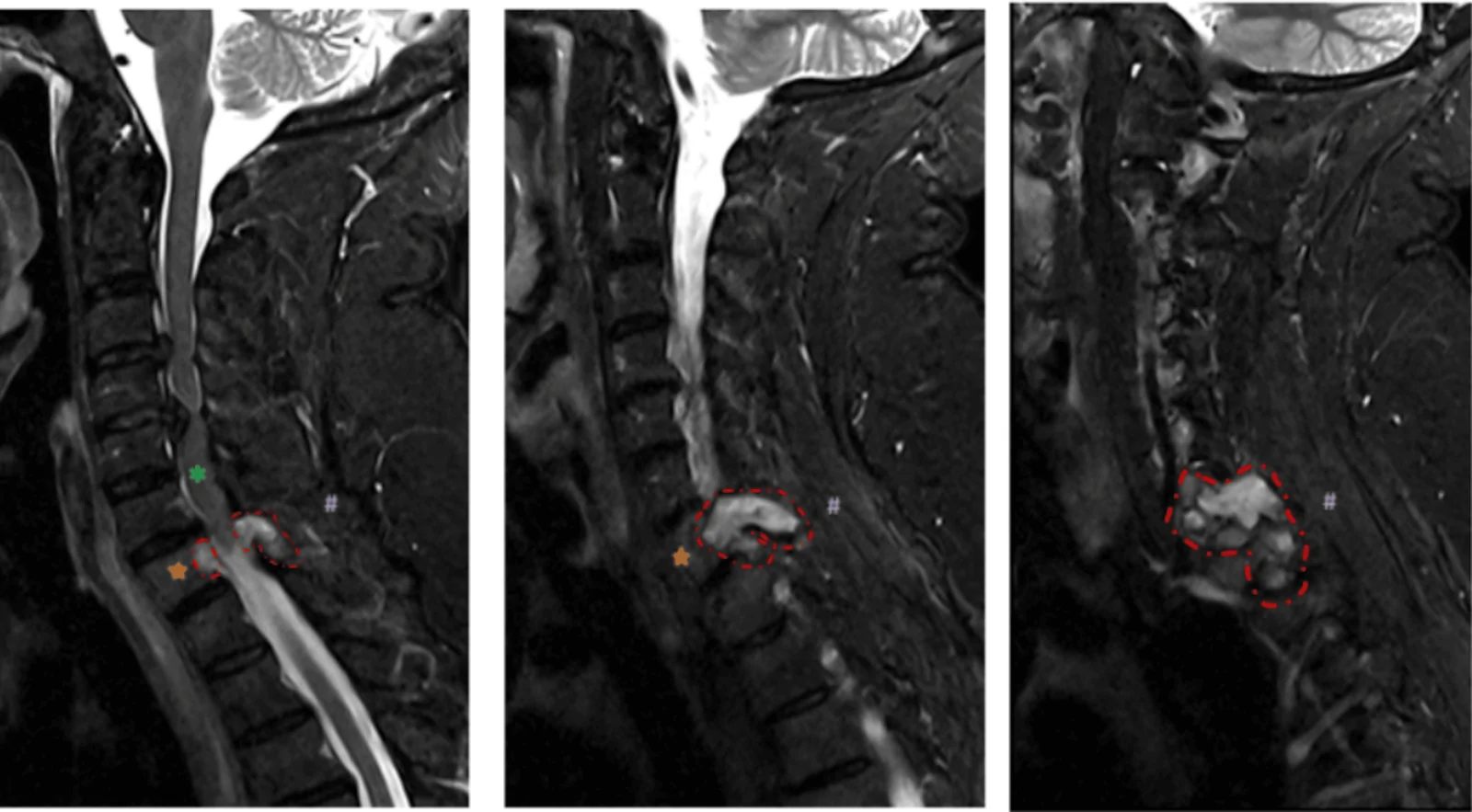
Cervical MRI demonstrating the localization of the FGF23-producing tumor at the C7 vertebral level MRI: magnetic resonance imaging; FGF23: FGF-23: fibroblast growth factor 23 Infiltrative lesion at the C7 vertebra with osseous destruction. The orange and green asterisks delineate the tumor margins, while the red circles indicate the tumor dimensions on different imaging planes

Transiliac bone biopsy demonstrated increased osteoid volume in cortical bone with preserved cortical thickness and porosity. Trabecular bone showed features of osteomalacia with impaired mineralization (Figure 1). A 68Ga-DOTATATE PET/CT scan performed in November 2022 demonstrated somatostatin receptor expression in a soft-tissue lesion involving the right aspect of the C7 vertebra. Cervical magnetic resonance imaging (MRI) confirmed an infiltrative lesion at the C7 vertebra with osseous destruction and extension into the epidural and paravertebral spaces, as well as into the C7-T1 neural foramen (Figure 2).

**Figure 3:**
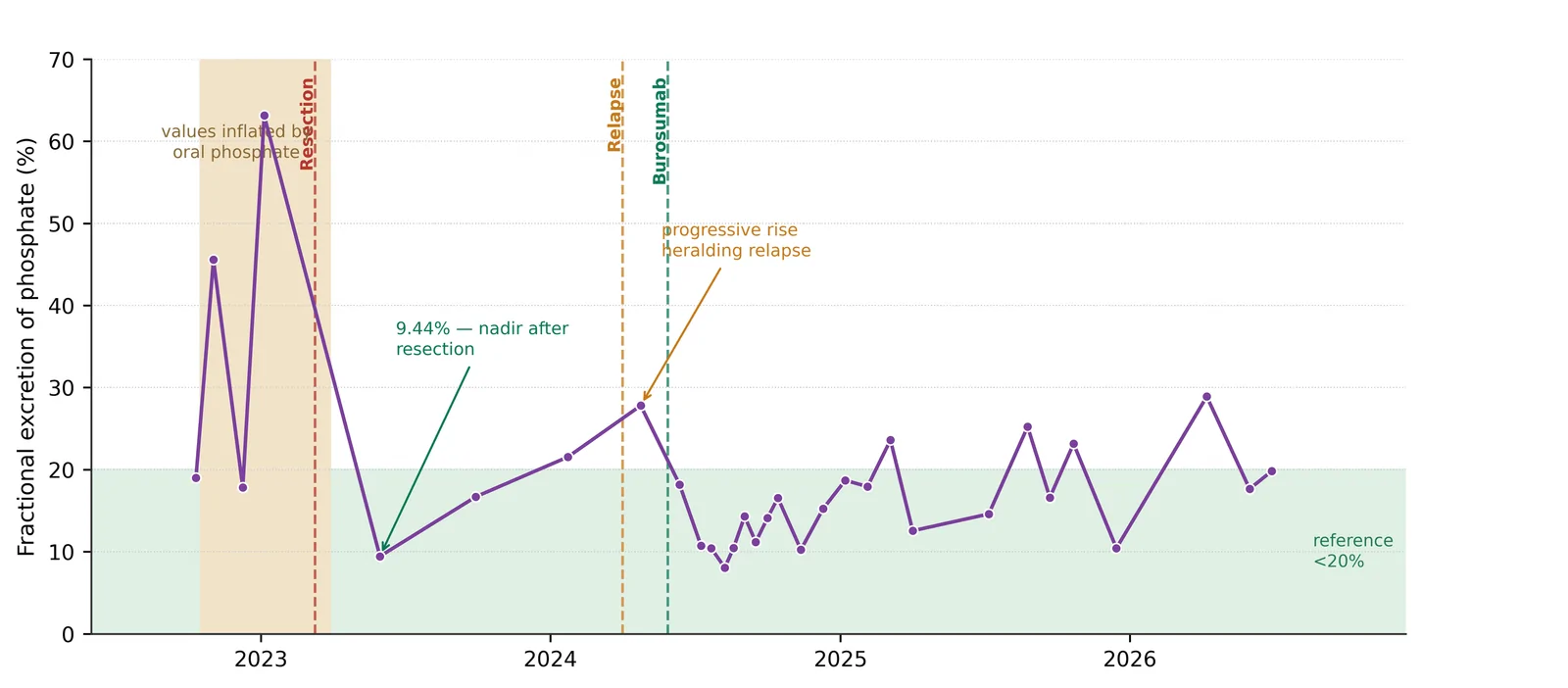
Fractional excretion of phosphate over time The horizontal shaded band indicates the expected fractional excretion of phosphate in the setting of hypophosphatemia (<20%). Values obtained during oral phosphate supplementation are artificially elevated and are indicated by the vertical shaded region

In March 2023, the patient underwent C7 laminectomy and pediculectomy with en bloc resection of the infiltrative lesion. The C6-C8 nerve roots were infiltrated by the tumor and required resection. Histopathological examination was inconclusive. Oral phosphate and calcitriol were discontinued three weeks after surgery. Serum phosphate normalized to 3.5 mg/dL, the FeP decreased to 9.44%, and the patient regained independent ambulation. At follow-up, serum phosphate remained within the low-normal range (2.5-2.7 mg/dL); however, FeP progressively increased from 16% to 21% (Figure 3), preceding symptomatic relapse.

In April 2024, recurrent bone pain and muscle weakness were accompanied by hypophosphatemia (2.2 mg/dL) and an increased FeP (27.8%). Repeat 68Ga-DOTATATE PET/CT demonstrated a new focus of somatostatin receptor uptake in the right parietal calvarium, whereas spinal magnetic resonance imaging showed no residual or recurrent cervical disease. As the calvarial lesion was not amenable to surgery or radiotherapy, burosumab, a human monoclonal antibody that inhibits FGF-23, was initiated at 40 mg every four weeks (0.51 mg/kg) in May 2024.

Biochemical parameters, including serum phosphate, calcium, PTH, 1,25-dihydroxyvitamin D, renal function, and FeP, were monitored every two weeks during the first six months of treatment and monthly thereafter. The patient was also monitored for treatment-related adverse events, including injection-site reactions and hypersensitivity, none of which occurred.

**Figure 4:**
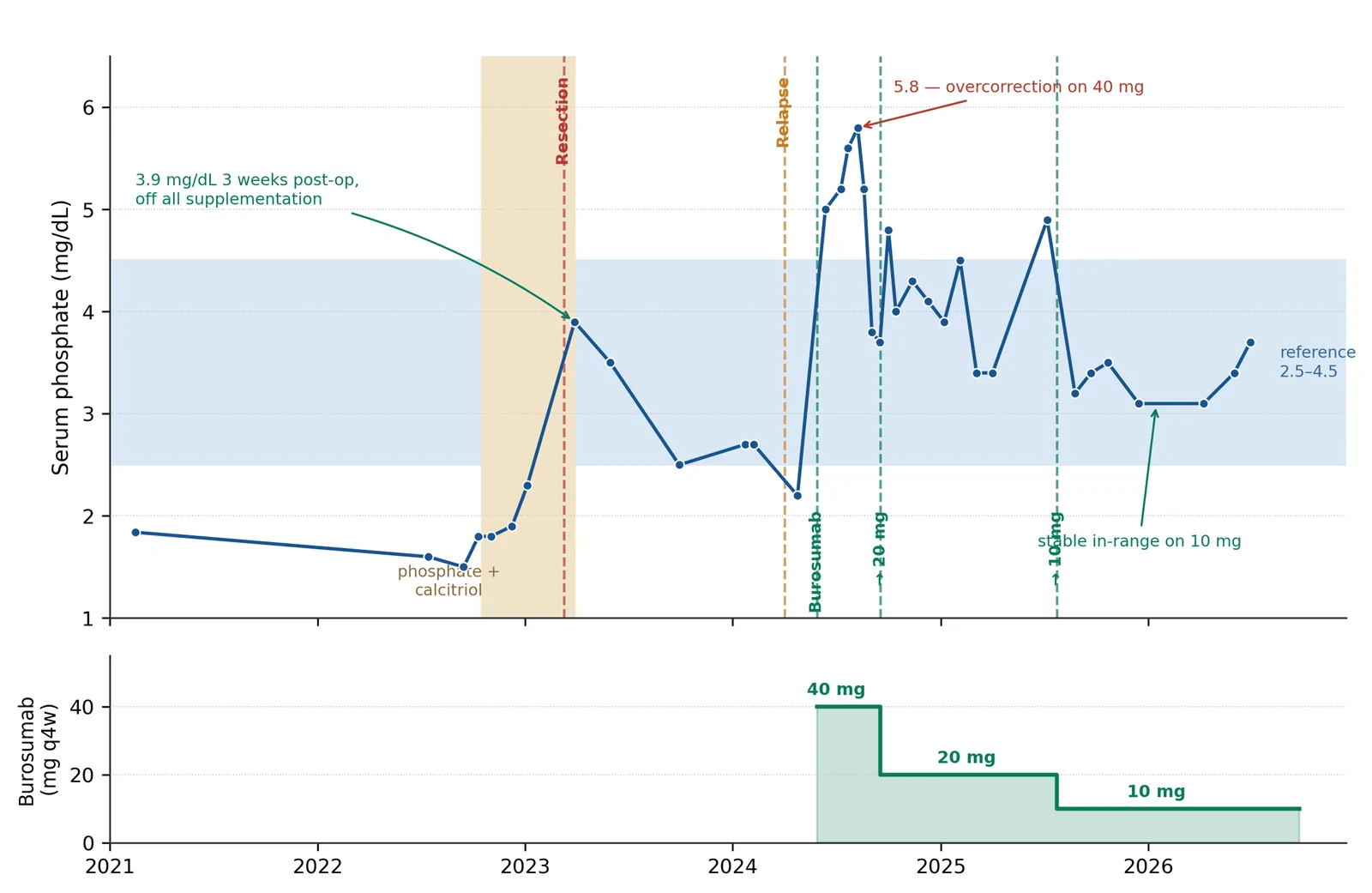
Serum phosphate concentrations throughout the different treatment phases, with corresponding burosumab doses The horizontal shaded area indicates the reference range (2.5-4.5 mg/dL), and the vertical shaded region denotes the period during which the patient received oral phosphate supplementation and calcitriol

Following transient hyperphosphatemia, the dose was reduced to 20 mg and subsequently to 10 mg every four weeks (Figure 4). Thereafter, the patient remained normophosphatemic and asymptomatic. FeP significantly declined after surgery and following burosumab initiation (Figure 3). The patient has since remained normophosphatemic and asymptomatic on the 10 mg regimen. After 25 months of treatment, serum phosphate was 3.7 mg/dL, and the patient remained independently ambulatory without requiring analgesics. Serial 68Ga-DOTATATE PET/CT scans demonstrated stable uptake in the right parietal calvarial lesion, with no evidence of disease progression. 

## Discussion

This case highlights three important aspects of the diagnosis and management of TIO. TIO remains a major diagnostic challenge because of its nonspecific presentation with bone pain, proximal muscle weakness, fatigue, and fragility fractures. Misdiagnosis rates approach 95%, with a median diagnostic delay of approximately three years, often resulting in progressive disability [9]. Our patient's clinical course closely reflected this pattern, having initially been diagnosed with osteoporosis and osteoarthritis before hypophosphatemia prompted further investigation. In adults presenting with unexplained musculoskeletal symptoms, systematic evaluation of hypophosphatemia is therefore essential [10]. Demonstration of renal phosphate wasting should prompt measurement of circulating FGF-23. Once FGF-23-mediated hypophosphatemia is established and hereditary disorders are excluded, localization of the responsible tumor becomes the priority. Combined functional and anatomical imaging, particularly 68Ga-DOTATATE PET/CT, has become the imaging modality of choice for tumor localization [11,12]. Although histopathological confirmation remains the diagnostic gold standard, the diagnosis in our patient was supported by characteristic biochemical abnormalities, concordant imaging findings, and normalization of phosphate metabolism following tumor resection.

This case illustrates that TIO may recur despite apparently curative surgery. Although complete tumor excision is curative in most patients, recurrence occurs in approximately 7-15% of cases, usually within the first three years after surgery [13,14]. Female sex, spinal location, bone involvement, malignancy, and low preoperative serum phosphate have been associated with an increased risk of recurrence [15]. Tumors involving the spine are exceptionally rare. To the best of our knowledge, 22 cases have been reported in the literature [8], and present considerable surgical challenges because of their infiltrative nature and proximity to critical neurovascular structures. In our patient, biochemical remission after cervical tumor resection was followed by recurrence within one year, emphasizing the need for continued biochemical and imaging surveillance even after apparently successful surgery.

In this context, burosumab provided an alternative to further treatment. Conventional treatment with oral phosphate and active vitamin D analogues is frequently limited by poor tolerability and long-term complications, including secondary hyperparathyroidism, hypercalciuria, nephrolithiasis, and nephrocalcinosis [16]. By neutralizing excess FGF-23, burosumab restores phosphate homeostasis without the need for chronic oral supplementation. Although it is not a tumor-directed therapy and does not replace complete surgical resection when feasible, previous reports have demonstrated its efficacy in patients with unidentifiable or unresectable TIO [17]. 

Day et al. described a patient with two somatostatin receptor-avid intracranial meningiomas in whom surgery was considered unsuitable; burosumab resulted in normalization of serum phosphate and improvement in pain and mobility [18]. Similarly, burosumab has been reported to normalize phosphate levels and improve pseudofractures, pain, and functional capacity in patients in whom extensive imaging and venous sampling failed to localize the responsible tumor [8]. Our case differs from these reports in that burosumab was used for recurrent disease after initially successful surgical resection, rather than as the initial definitive treatment. Furthermore, the patient achieved sustained biochemical and clinical control for 25 months despite persistent uptake of the recurrent calvarial lesion on serial 68Ga-DOTATATE PET/CT. Of note, there were no new fragility fractures or treatment-related adverse events.

To our understanding, this is the first reported case of TIO in Portugal successfully treated with burosumab. These findings are consistent with prospective clinical studies and published case reports demonstrating durable biochemical correction and improvements in physical function and health-related quality of life [19]. Nevertheless, continued biochemical and imaging surveillance remains essential to monitor disease progression.

## Conclusions

In conclusion, this case adds to the growing evidence supporting burosumab as an effective long-term treatment for unidentifiable or unresectable TIO. However, data on prolonged therapy remain limited, and the long-term effects of sustained FGF-23 inhibition on bone health, renal function, ectopic calcification, and disease progression are not yet fully established. Further longitudinal studies are needed to better define the long-term efficacy and safety of burosumab in patients with TIO.
